# Changes of B cell subsets in central pathological process of autoimmune encephalomyelitis in mice

**DOI:** 10.1186/s12865-019-0301-4

**Published:** 2019-07-08

**Authors:** Yingqiong Xiong, Shaomin Cheng, Xiaomu Wu, Yue Ren, Xufang Xie

**Affiliations:** 1 Graduate School, Nanchang University, Nanchang, China; 2 Department of Neurology, Jiangxi People’s Hospital, 153 Aiguo road, Nanchang, China; 3 Institute of Neurology, Jiangxi People’s Hospital, Nanchang, China; 4 Key Laboratory, Department of Neurology, Jiangxi People’s Hospital, Nanchang, China; 5 School of Basic Medical Sciences, Jiangxi University of traditional Chinese Medicine, Nanchang, China

**Keywords:** Multiple sclerosis, B cell, EAE, TPI, GADPH

## Abstract

****Background**:**

Multiple sclerosis is a demyelinating and autoimmune disease and its immune response is not fully elucidated. This study was conducted to examine the pathological changes and B cell subsets in experimental autoimmune encephalomyelitis (EAE) mice, and analyze the expression of triosephosphate isomerase (*TPI*) and GADPH to define the role of B cell subsets in the disease.

**Results:**

Female C57BL/6 mice were randomly divided into EAE group (*n* = 18) and control (*n* = 18). During the experiments, the weight and nerve function scores were determined. The proportions of B cell subsets in the peripheral blood were measured by flow cytometry. Seven, 18 and 30 days after immunization, the brain and spinal cord tissues were examined for the infiltration of inflammatory cells using *he*matoxylin-eosin (HE) HE staining and the demyelination using Luxol fast blue staining. The expression of B cell-related proteins was detected immunohistochemistrially and the expression of antigenic TPI and GADPH was analyzed using enzyme-linked immunosorbent assay (ELISA). HE staining showed that mice had more severe EAE 18 d than 7 d after modelling, while the symptoms were significantly relieved at 30 d. The results were consistent with the weight measurements and neural function scores. Immunohistochemistry studies showed that B cells aggregated in the spinal cord, but not much in the brain. Flow cytometry studies showed that there were more B cells in control than in EAE models from day 7 and the difference was narrowed at day 30. The level of plasma cells increased continuously, reached the top at day 21 and obviously declined at day 30. On other hand, the numbers of memory B cells increased gradually over the experimental period. The numbers of plasma and memory B cells were similar between the control and EAE mice. ELISA data revealed that the brain contents of TPI and GAPDH were higher in EAE mice than in control at day 7, while at day 18, the levels were reversed.

**Conclusions:**

In the central pathological process of EAE mice, B cells exert role through the mechanism other than producing antibodies and the levels of brain TPI and GADPH are related to the severity of autoimmune induced-damage.

## Background

Multiple sclerosis (MS) is a demyelinating disease in the white matter of the central nervous system. It is an autoimmune disease associated with genetic and environmental factors in susceptible individuals. A variety of immune cells, cytokines, antibodies and complements are involved in MS that leads to the destruction of oligodendrocyte and myelin in the axon and demyelination [1] The study of MS pathogenesis is main

ly based on animal models of autoimmune encephalomyelitis (EAE) induced by autoimmunization of myelin basic protein (MBP) [2, 3], in which human MOG35–55 is used as an antigen mimicry to generate immune response to attack the mouse nerve cells.

Recent studies have found that during the pathogenesis of MS, B cells play an important role. For example, when MS was treated with Rituximab depleted CD20^+^ cells, colony stimulating factor (CSF) and serum B cells were greatly decreased, leading to low clinical recurrence, reduced intracranial inflammation and better curative effect [4] . One of the major functions of B cells is to produce antibodies, and in some MS patients pathogenic antibodies were identified at the lesions [5]. However, other studies have also found that the level of antibody in the plasma cells and cerebrospinal fluid in MS patients is basically unchanged after receiving B cell depletion therapy, and B cells may play role in MS via other mechanisms [6]. In the EAE model, MHC-II–deficient B cells can produce autoantibodies but can not stimulate T cells to generate EAE, indicating that the antigen presentation of B cells is very important to myelin oligodendrocyte glycoprotein (MOG)-induced EAE [7]. TPI is mainly distributed in the neurons and axons of the central nervous system. When the activity of the enzyme is disturbed, it can cause neuron and axon degeneration. It has been shown that in many autoimmune diseases TPI antibody is increased [8]. 3- glyceraldehydehydrogenase (GAPDH) was originally thought to play a role as oxidoreductase in the cytoplasm, but its new functions are continuously discovered. The antibodies and the single-strand variable fragment antibodies in the cerebrospinal fluid of MS patients were immunoreactive to GAPDH, suggesting that GAPDH could promote the proliferation of B cells in the central nervous system in MS patients [9, 10].

In this experiment, we analyzed the pathological changes and B cell subsets in the EAE mice and determined the content of brain TPI and GAPDH in order to elucidate the pathogenic mechanism of MS.

## Results

### Weight and nerve function score

**Fig. 1:**
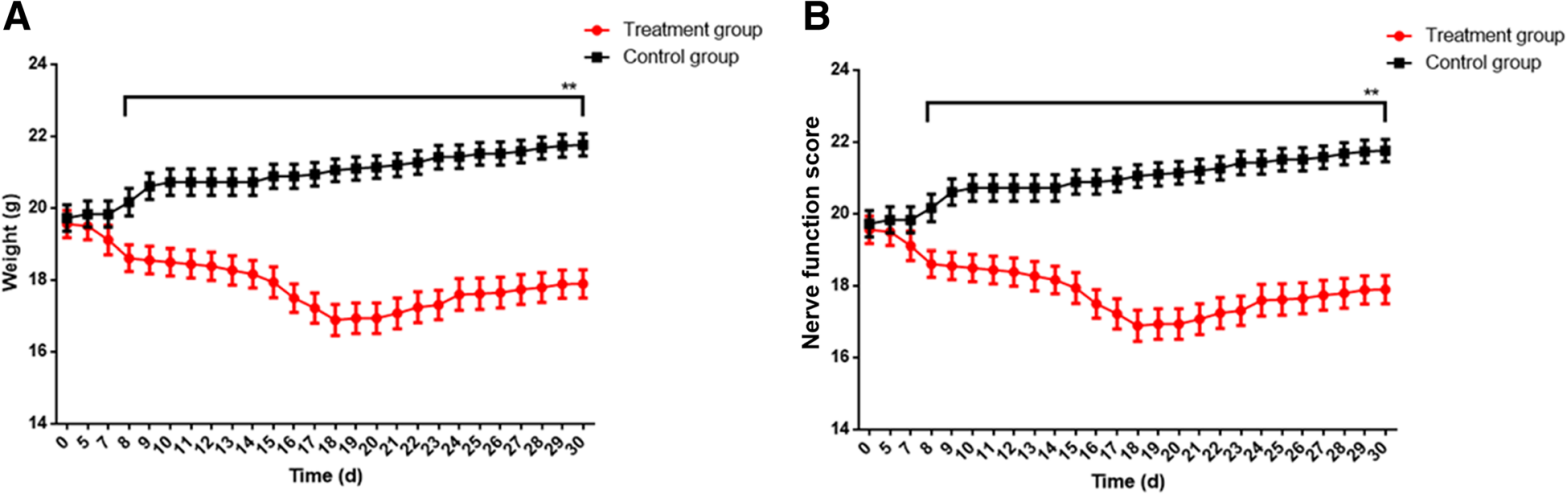
Weights (**a**) and nerve function scores (**b**) of EAE mice over 30 day experimental period. ** denotes *P* < 0.01 vs control

The weights of EAE mice was significantly lighter than those of the control mice, and the difference in weight gradually increased from the beginning of modelling, reached the peak in about 20 days, and then narrowed slightly (Fig. 1a). The scores of nerve functions increased gradually, reached the peak on day 18 with a score of about 3 and then then gradually declined with a score of 1 at day 30 (Fig. 1b).

### Changes in B cell subsets

**Fig. 2:**
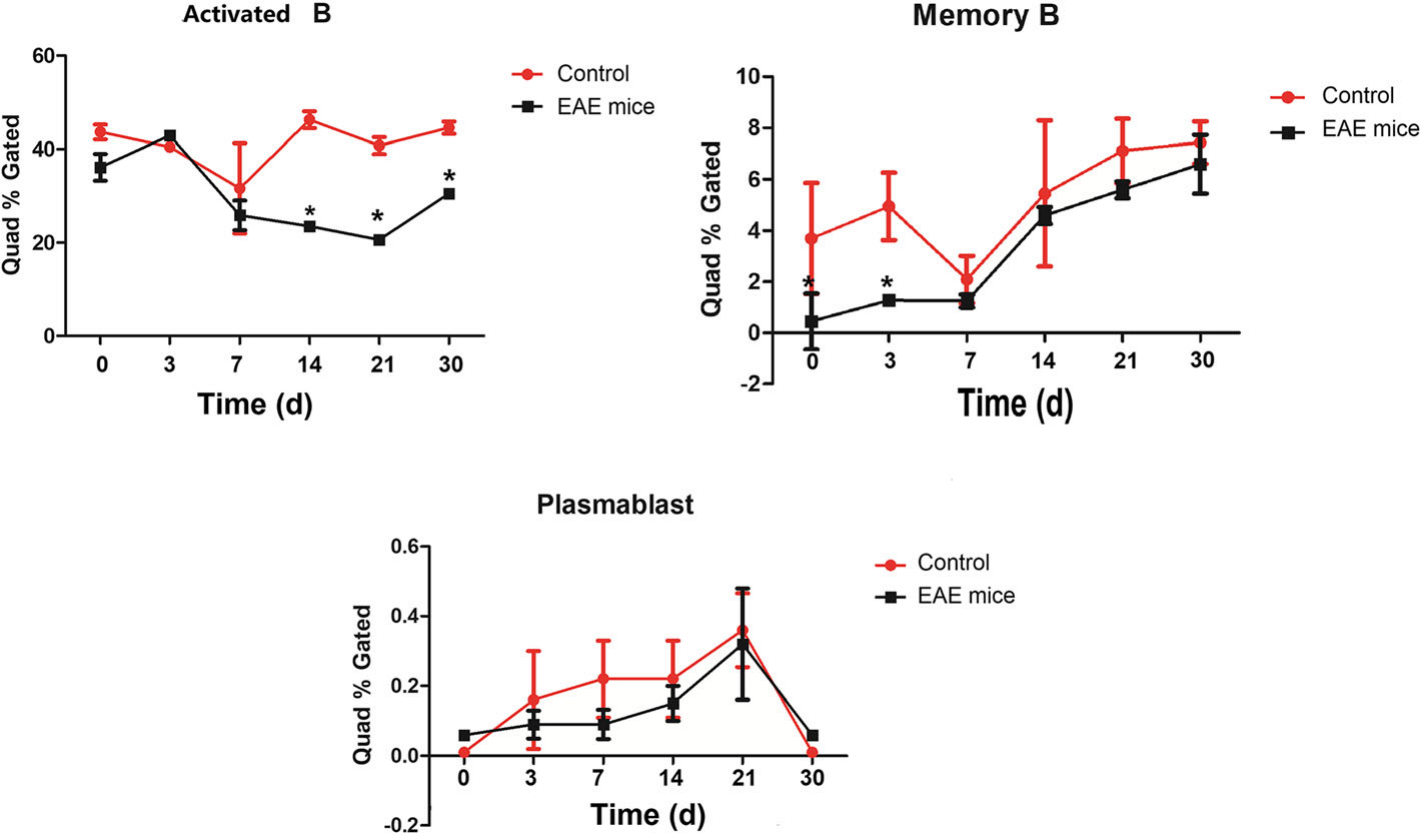
Changes in B cell subsets in EAE mice over 30 day experimental period. The activated B-cells, memory B-cells and plasma cells were determined using markers CD19^+^ and IgD^+^(activated B-cell), CD19^+^, CD27^+^ and B220^+^ (memory B-cell) and CD19^−^and CD138^+^ (plasma cell).* denotes *P* < 0.05 vs control

Flow cytometry showed that the percentage of activated B cells in peripheral blood decreased from the 7th day, and was significantly lower in EAE group than in control group from the 14th day. However, the difference narrowed on the 30th day. The percentage of memory B cells in EAE group was lower than that of normal group at the beginning of modeling, and the difference began to narrow from the 7th day, and varnished afterwards. The level of plasma cells increased continuously, and reached the highest value on the 21st day, and decreased significantly on the 30th day. However, there was no significant difference between the groups the experimental period (Fig. 2).

### Pathological changes

**Fig. 3:**
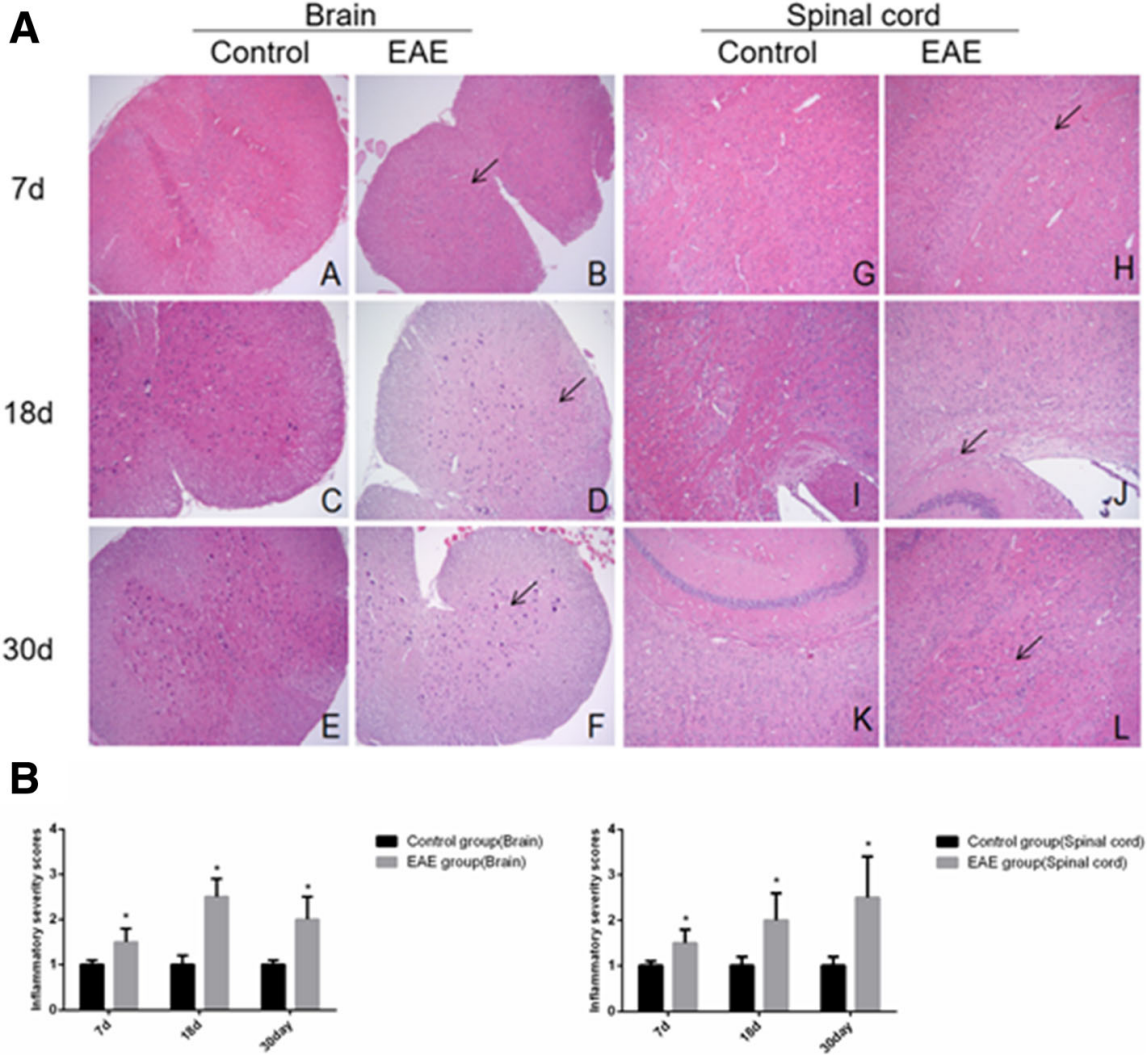
HE staining (**a**) and inflammatory severity score (**b**) of brain and spinal cord tissues of EAE mice (× 100). A and G. control at day 7; B and H, EAE mice at day 7; C and I, control at day 18; D and J, EAE mice at day 18, E and K. control at day 30 and F and L. EAE mice at day 30, respectively. A-F, spinal cord tissue; G-L, brain tissue. Arrows denote inflammatory cells. * denotes *P* < 0.05 vs control

HE staining showed that in EAE mice, there were a large number of inflammatory cells infiltrated into the parenchyma of the spinal cord and some white matters were demyelinated and stained as vacuoles (Fig. 3a). The control mice were intact with significantly fewer inflammatory cells. Similar results were observed in the brain tissue (Fig. 3b).

### Demyelination of myelin

**Fig. 4:**
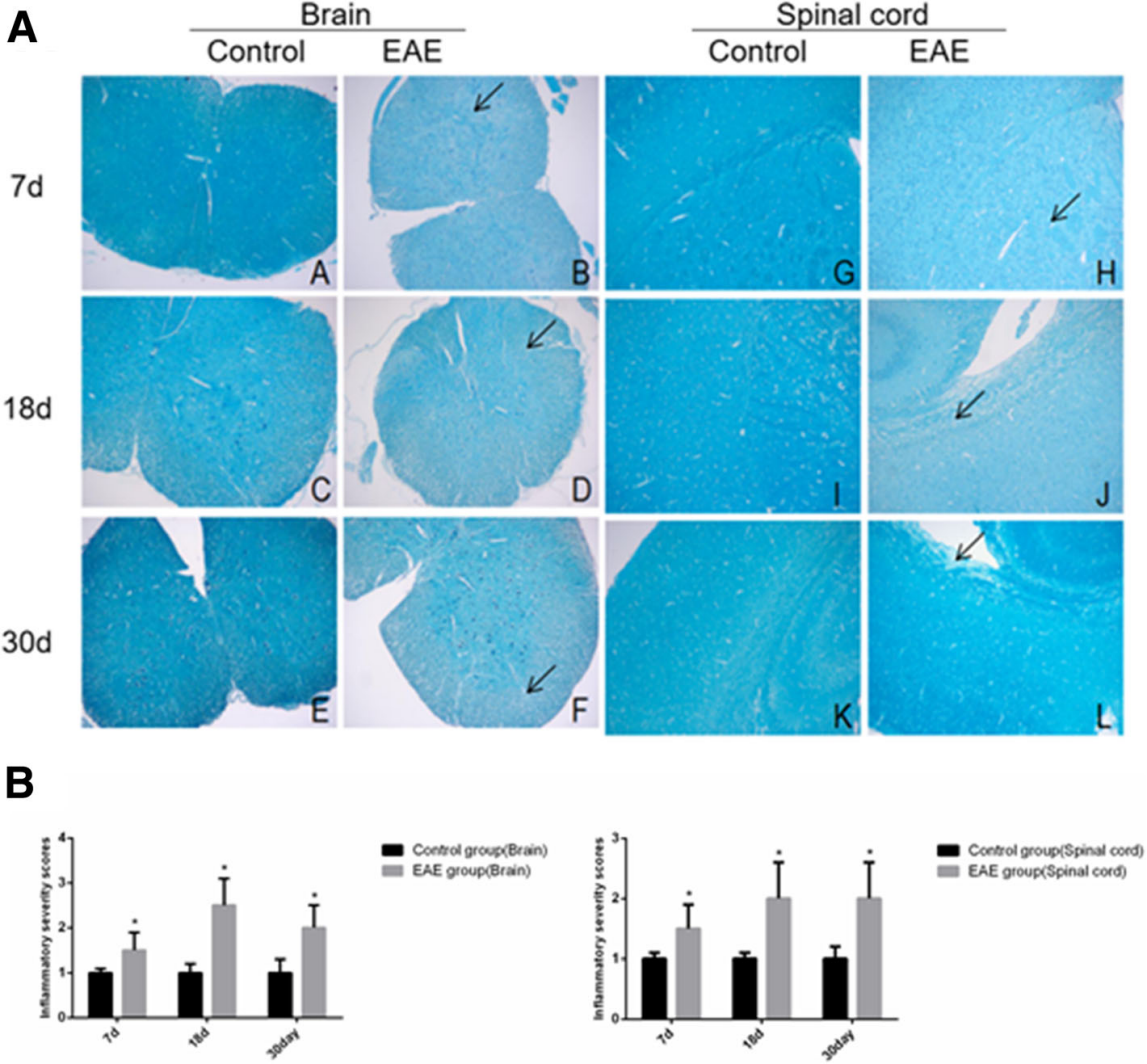
LFB staining (**a**) and inflammatory severity score (**b**) of brain and spinal cord tissues of EAE mice (× 100). A and G. control at day 7; B and H, EAE mice at day 7; C and I, control at day 18; D and J, EAE mice at day 18, E and K. control at day 30 and F and L. EAE mice at day 30, respectively. A-F, spinal cord tissue; G-L, brain tissue. Arrows denote demyelinated myelin. * denotes *P* < 0.05 vs control

LFB staining showed that a large number of myelins in the white matter around the spinal cord and brains of EAE mice were demyelinated and were unstained compared with the control mice, (Fig. 4a). The control mice were intact with significantly fewer inflammatory cells. Similar results were observed in the brain tissue (Fig. 4b).

### Expression of B cell subset-related markers

**Fig. 5:**
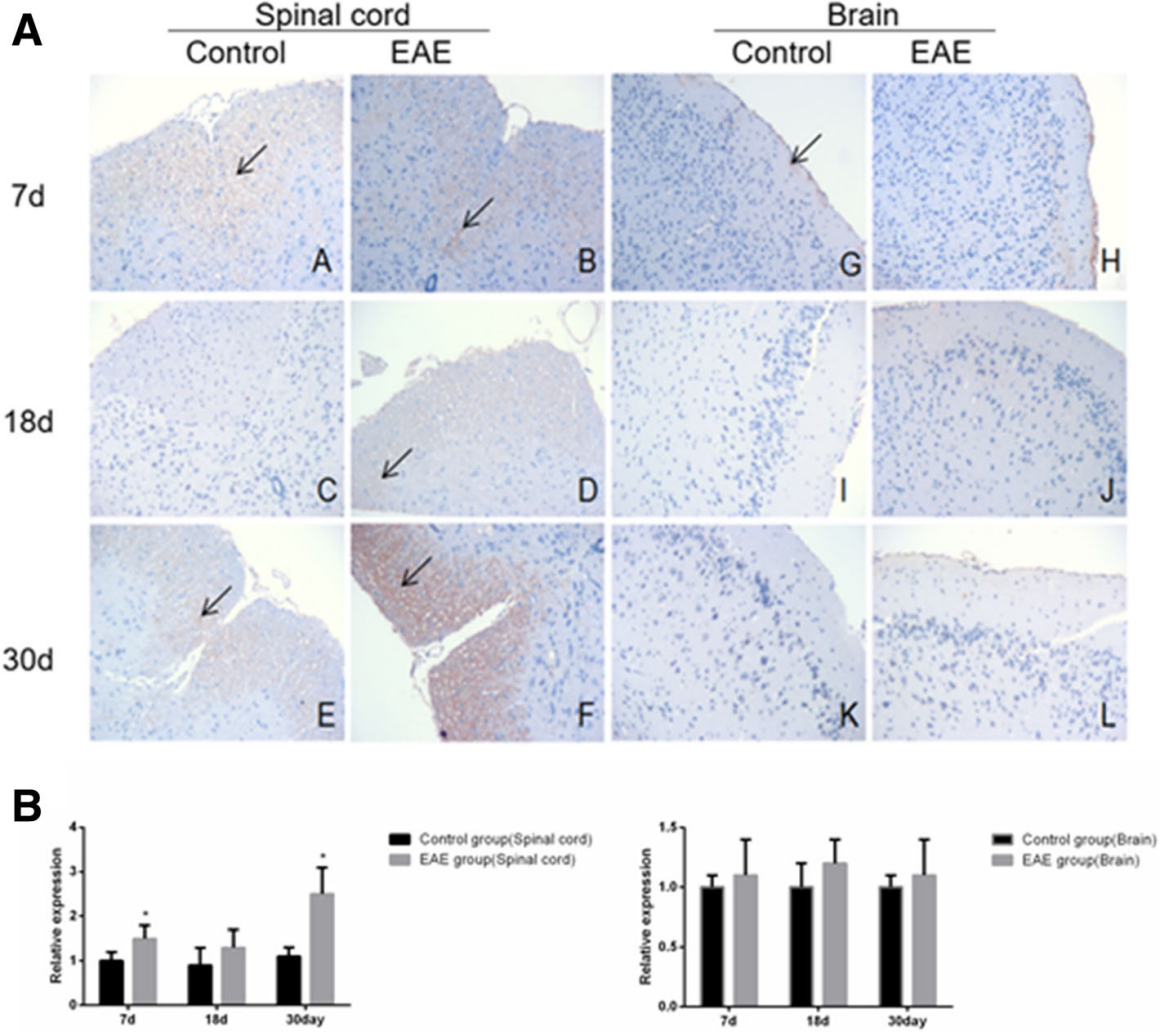
Immunohistochemical assays (**a**) and relative expression (**b**) of CD19^+^ cells in the spinal cord and brain tissue of EAE mice (× 200). A and G. control at day 7; B and H, EAE mice at day 7; C and I, control at day 18; D and J, EAE mice at day 18, E and K. control at day 30 and F and L. EAE mice at day 30, respectively. A-F, spinal cord tissue; G-L, brain tissue. Arrows denote CD19^+^ cells. * denotes *P* < 0.05 vs control

**Fig. 6:**
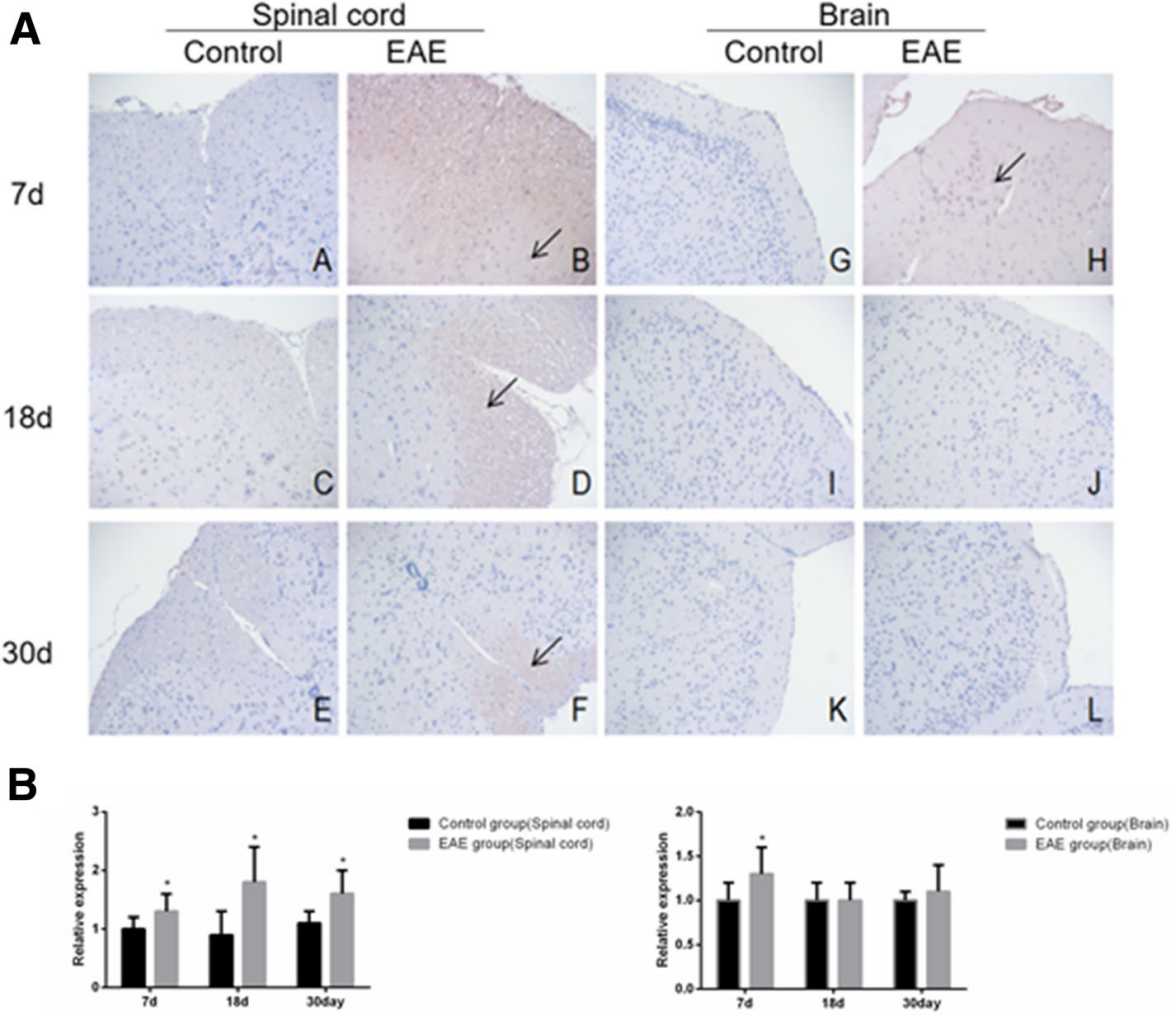
Immunohistochemical assays (**a**) and relative expression (**b**) of IgD^+^ cells in the spinal cord and brain tissue of EAE mice (× 200). A and G. control at day 7; B and H, EAE mice at day 7; C and I, control at day 18; D and J, EAE mice at day 18, E and K. control at day 30 and F and L. EAE mice at day 30, respectively. A-F, spinal cord tissue; G-L, brain tissue . Arrows denote IgD^+^ cells. * denotes *P* < 0.05 vs control

**Fig. 7:**
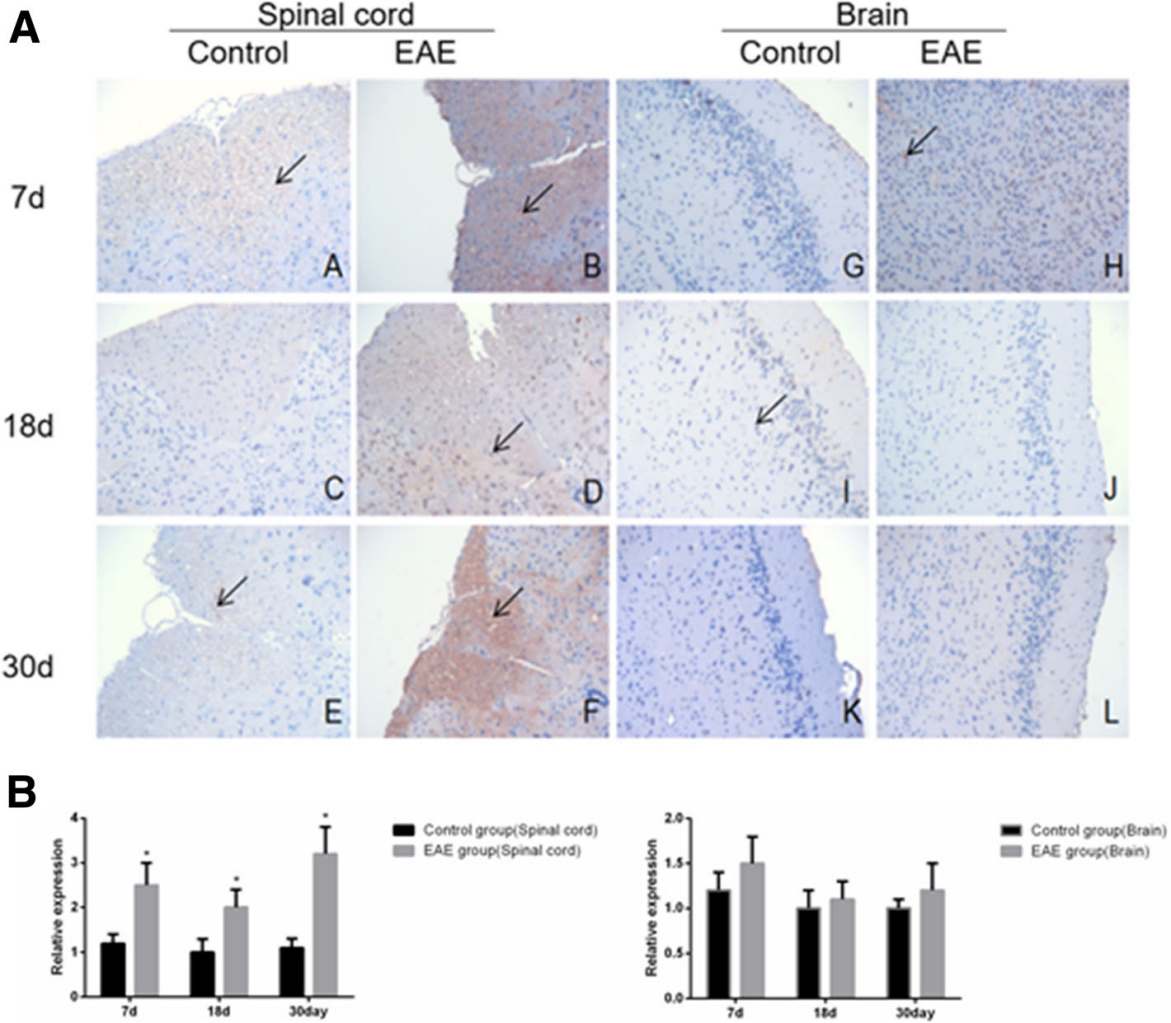
Immunohistochemical assays (**a**) and relative expression (**b**) of CD27^+^ cells in the spinal cord and brain tissues of EAE mice (× 200). A and G. control at day 7; B and H, EAE mice at day 7; C and I, control at day 18; D and J, EAE mice at day 18, E and K. control at day 30 and F and L. EAE mice at day 30, respectively. A-F, spinal cord tissue; G-L, brain tissue. Arrows denote CD27+^+^ cells. * denotes *P* < 0.05 vs control

**Fig. 8:**
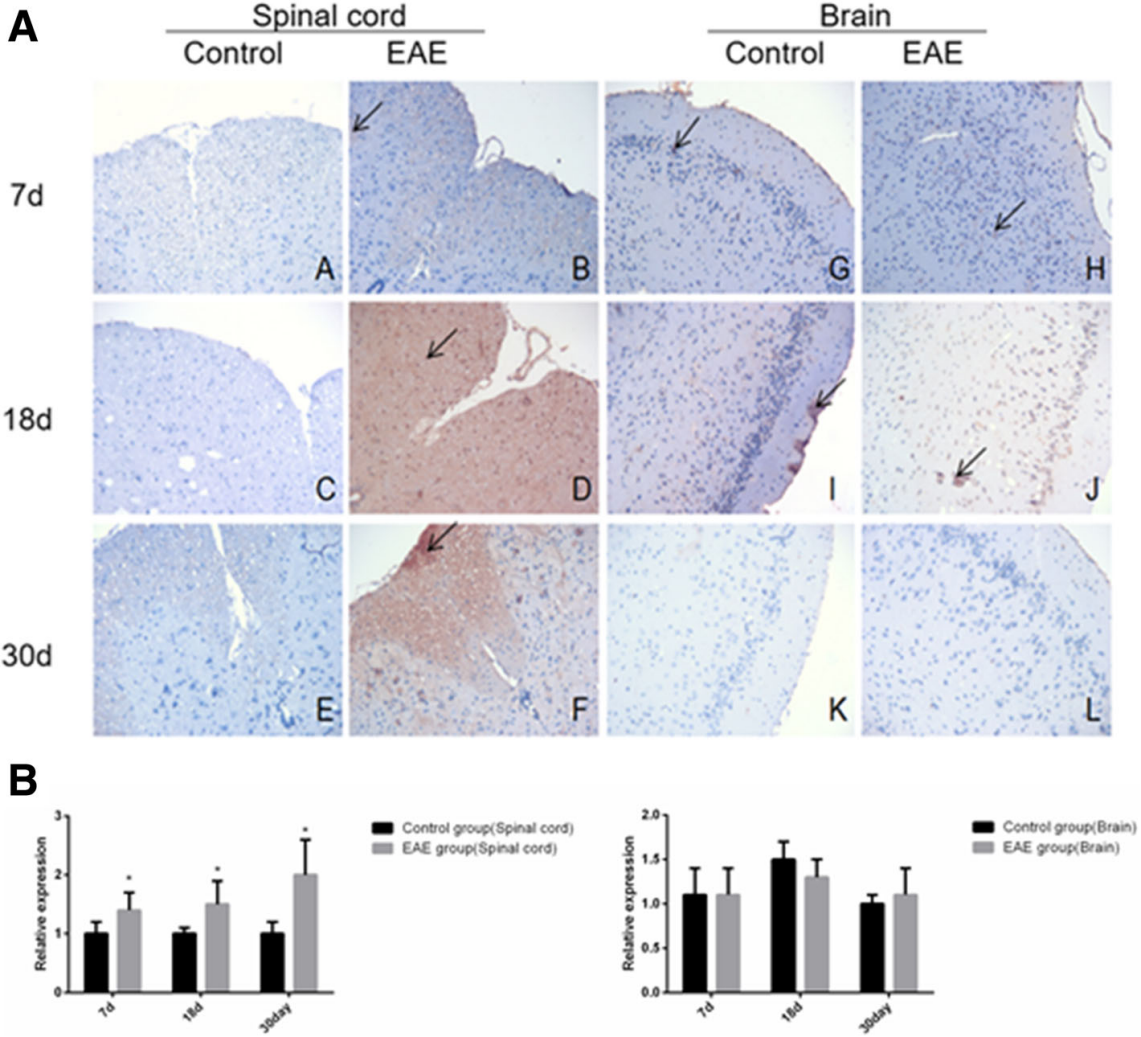
Immunohistochemical assays (**a**) and relative expression (**b**) of B220^+^ cells in the spinal cord and brain tissue of EAE mice (× 200). A and G. control at day 7; B and H, EAE mice at day 7; C and I, control at day 18; D and J, EAE mice at day 18, E and K. control at day 30 and F and L. EAE mice at day 30, respectively. A-F, spinal cord tissue; G-L, brain tissue. Arrows denote B220^+^ cells. * denotes *P* < 0.05 vs control

**Fig. 9:**
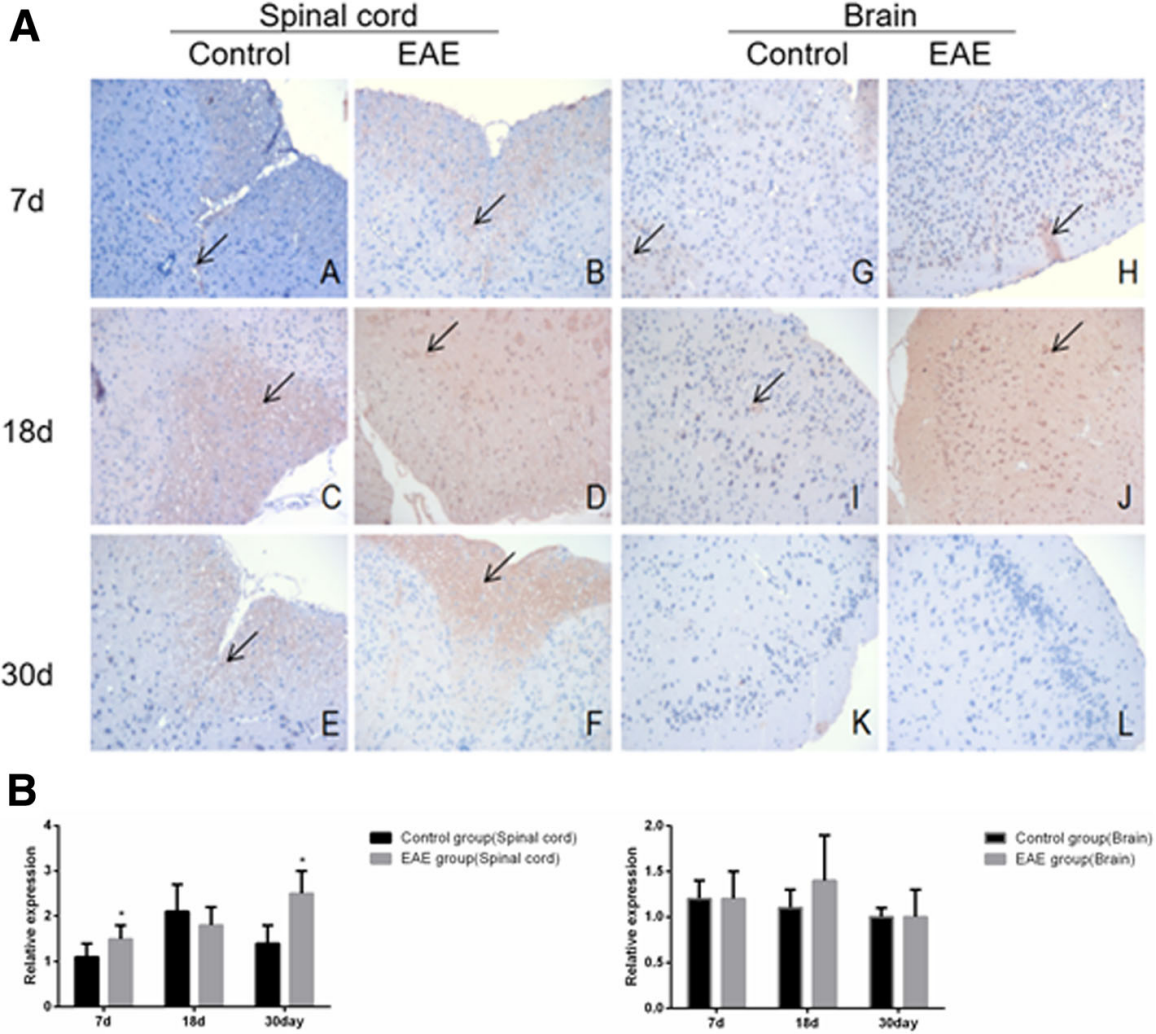
Immunohistochemical assays (**a**) and relative expression (**b**) of CD138^+^ cells in the spinal cord and brain tissue of EAE mice (× 200). A and G. control at day 7; B and H, EAE mice at day 7; C and I, control at day 18; D and J, EAE mice at day 18, E and K. control at day 30 and F and L. EAE mice at day 30, respectively. A-F, spinal cord tissue; G-L, brain tissue . Arrows denote CD138^+^ cells. * denotes *P* < 0.05 vs control

Immunohistochemical assays showed that the expression of B cell subset -related markers CD19^+^, IgD^+^, CD27^+^, B220^+^ and CD138^+^ was higher in the spinal cord, but similar in the brain tissues of EAE mice than in control (Fig. 5, 6, 7, 8 and 9).

### Content of TPI and GAPDH

**Fig. 10:**
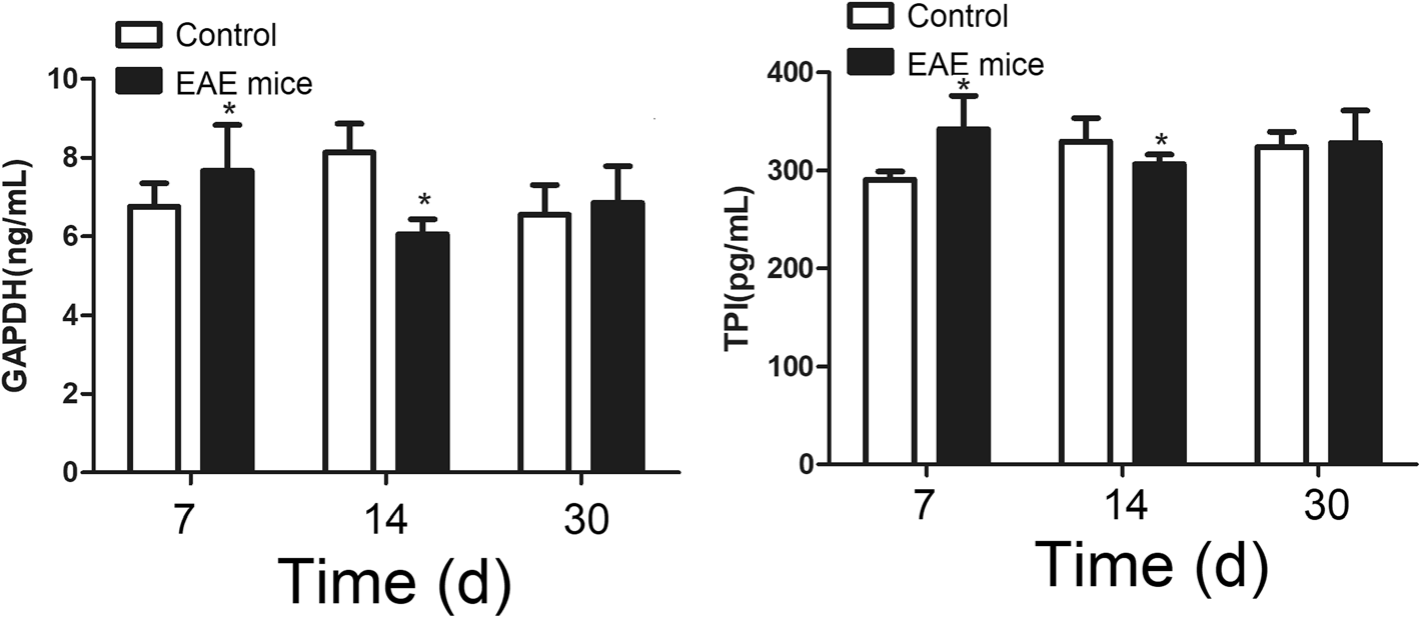
Content of cerebrospinal fluid TPI and GAPDH of EAE mice. * denotes *P* < 0.05 vs control

ELISA results showed that the contents of cerebrospinal flui TPI and GAPDH were significantly higher (*P* < 0.05) in EAE mice than in control on days 7, but the contents were significantly lower in EAE mice than in control on day 14 (Fig. 10).

## Discussion

For a long time, MS is mainly recognized as a T cell-mediated autoimmune disease [11]. However, recent studies show that not only B cell-mediate humoral immunity is involved in the pathogenesis of the disease, but also the cytokine secreted by B cells play regulatory role in T cell response [12]. Moreover, there are heterotopic proliferative lymphoid follicle of B cells in the meninges of MS patients, and B cells, plasma cells, complements in the active lesions [13]. The differentiation of B lymphocytes starts from bone marrow hematopoietic stem cells and goes through progenitor B cells, pre B cells, immature B cells, initial B cells, activated B cells, memory B cells, and plasmablasts to plasma cells that secrete antibodies [14]. It is known that functional abnormalities of initial B cells and memory B cells can lead to a variety of human autoimmune diseases [15]. Therefore, we analyzed the changes of these two subsets as well as the production of the final effector cells, plasma cells.

HE staining shows that EAE mice had more brain tissue and spinal cord damage at day 18 than at day 7, but much less at day 30.These findings are consistent with the weight measurements. These data reveal that the symptoms of EAE mice aggravated till about 3 weeks after MOG immunization. Once stimulated by antigens, initial B cells may develop in the bone marrow through the progenitor B cells, activated B cells and immature B cells may differentiate into mature B cells in the peripheral lymphoid organs. If there is an antigen stimulation, the initial B lymphocyte can develop in the lymphatic follicle to form germinal center B cells [16]. The peripheral blood data are results obtained from the myelitis part of encephalomyelitis. Since pertussis was injected during modelling, the permeability of the blood brain barrier is increased, promoting the entrance of the immune cells, cytokines and antibodies in the peripheral blood into the brain [17]. However, immunohistochemical assays show that B cells were accumulated in the spinal cord of EAE mice, but not much in the brain. In the peripheral blood, more activated B cells appeared at day 7 in the control and in EAE mice, and the difference narrowed at the end of experiment. The amount of plasma cells increased continuously till day 18 and then declined. The amount of activated B cells began to decline after immunization and was lower in EAE mice than in control, suggesting that in EAE mice activated B cells may be mobilized to accumulate in the germinal center of lymphoid organs from the early stage of disease, leading to reduced amount in the peripheral blood. Therefore, we speculate that the activated B cells are involved in the autoimmunization process in the EAE mice. The percentage of memory B cells in EAE group was lower than that of normal group at the beginning of modeling, and the difference began to narrow from the 7th day, and varnished afterwards.. It has been found that the ratio of CD19^+^ IgD^+^ activated B cells in spleen and lymph tissue of EAE mice increased significantly at the peak of disease [14].

On other hand, the impact of plasma and memory B cells, which are associated to the production of antibodies, on the autoimmunization process in EAE mice is rather limited. TPI and GAPDH are important enzymes in the process of energy metabolism. We speculated that TPI and GAPDH in the cerebrospinal fluid can reflect the number of cells (mainly nerve cells) damaged and the amount of intracellular proteins released into the cerebrospinal fluid [8, 9]. Based on previously studies, TPI and GAPDH are important antigens in the EAE model that generate antibodies in the autoimmune response. The expression of TPI antibody was also shown to be associated to Expanded Disability Status Scale (EDSS) scores only in MS patients but not in non-MS patient [17]. It has been found that single-chain variable fragments antibodies in the cerebrospinal fluid of MS patients is immunoreactive to GAPDH, and the anti-GAPDH immunoglobulin G inhibits the glycolytic activity of GAPDH [8, 11]. We therefore speculated that if the content of TPI and GAPDH in cerebrospinal fluid is high, there would be more B cells that produce corresponding antibodies as a result of positive feedback. We find that the contents of TPI and GADPH in the cerebrospinal fluid were higher in EAE mice than in control on day 7 and lower in EAE mice than in control on day 18. This is consistent with early work that the expression of TPI antibody is increased in the brain lesions and the spinal fluid of MS patients [7]. Based our data, after initial increase, the contents of TPI and GAPDH were even slightly lower than those in the control group when encephalomyelitis reached its peak 2 weeks later, indicating that cell damage is controlled after the initial stage, but the inflammation reaction would continue to develop for some time. As such, TPI and GAPDH may not be the key autoimmune antigen. It is premature to judge whether TPI and GAPDH can promote EAE-related autoimmune response. However, it is very likely that in the EAE mice, TPI and GAPDH may be involved in severe early inflammatory response, and their roles are reduced as the nerve cells are damaged and their amounts are correspondingly reduced.

## Conclusions

It is likely that that the B cells play a role in the central pathological process in EAE mice mainly through the mechanisms other than producing antibodies, and the amounts of brain TPI and GADPH are related to the severity of damage induced by autoimmunization.

## Methods

### Experimental animals

Special pathogen-free C57BL/6 mice, female, 6–8 weeks old, weighting 18–20 g, were obtained from Experimental Animal Center, Military Medical Science Academy of People’s Liberation Army, Nanjing, China (permit no. NCSK 2016–0012). All animal experimental protocols were approved by the ethics committee for animal care and study at Jiangxi People’s Hospital, China. Mice that survived after the completion of the experiments were sacrificed by CO_2_ asphyxiation.

### Reagents and equipment

Complete Freund adjuvant (CFA, batch number: #SLBR3879) and Pertussis (batch number #SLBRV6619) were purchased from Sigma, USA. Rabbit antibodies against mouse CD19 (bs-4755R, 1:200), mouse CD4 (bs-0647R, 1:250) and mouse CD45 (bs-10599R, 1:250) were purchased from Boosen, Beijing China. FITC-labeled antibody against mouse CD19 (11–0193-82) was purchased from eBioscience, USA. PE- labeled antibodies against mouse IgD (405705), mouse CD27 (124209) and mouse CD138 (142506) were purchased from Biolegend, USA. APC-labeled antibody against B220 (553092) was purchased from BD Biosciences, USA. Mouse TPI and GAPDH ELISA detection kit were obtained from Mlbio, Shanghai, China. Flow cytometry (NovoCyte 2060R) was a product of BD, USA.

### Experimental grouping

Mice were randomly divided into control and EAE groups (*n* = 18). EAE mice were generated by injection of MOG35–55 to induce autoimmunization as reported previously [18], and the control animals were treated with normal saline. All animals were reared under the same environmental conditions. Mice that survived the experiments were sacrificed by CO_2_ asphyxiation after completion of experiments.

### EAE modelling

Mice were immunized using MOG 35–55 polypeptide with CFA. The antigen MOG35–55 was diluted into 300 μg/ml in 0.01 mol/l PBS and mixed with equal amount of CFA. The mixture was emulsificated and 0.2 ml antigen emulsion was injected subcutaneously on both sides of the ventral midline of the spinal cord into mice, which were anaesthetized by injecting intraperitoneally with 0.6% pentobarbital sodium at 70 mg/kg. 48 h after immunization, 500 ng pertussis was injected into the abdominal cavity. The models began to show the symptoms of impairments 7 days after modelling, which reached a peak in about 20 days. After that, the mice were gradually recovered. By the end of the experiment, the mice were fully recovered.

### Weighting and nerve function scoring

From the day of modelling, the mental state and activity of mice were observed and recorded daily. The animals were weighted and scored for neurological functions as described previously [10, 19, 20]. EAE Score 0 for no paralysis; 1 for flaccid tail; 2 for moderate hind-limp paralysis; 3 for complete hind-limp paralysis; 4 for fore-limb paralysis; and 5 death.

### Blood and tissue sampling

Peripheral blood was taken from the clump of veins at the eye sockets of the mice 0, 3, 7, 14, 21 and 30 days of immunization. Cerebral spinal cord was taken 7, 18 and 30 days after the immunization. Before taking the cord, the mice were anaesthetized, abdominal cavity opened, injected subcutaneously with 5 ml normal saline. While injecting normal saline, the right atrial appendage was cut and injected with 10% formalin to fix. Isolated cerebral spinal cord was stored in centrifuge tube containing 10% formalin at 4 °C. The cerebrospinal fluid was taken from anaesthetized and scarified mice and stored in centrifuge tube at − 20 °C.

### Flow cytometry

Peripheral bloods were suspended in PBS, labeled with FITC-, or PE-labeled antibodies against CD3, CD4, CD19, CD25, CD27, B220 and CD138CD19 following the manufacturer’s instruction. After gentle mixing, the suspensions were incubated for 20 min at room temperature in the dark. The cells were pelleted after centrifuging at 2300 rpm for 1 min and the pellets were added with 1 mL membrane breaking agent, vortexed and incubated for 13 min in the dark. The cells were pelleted after centrifuging at 2300 rpm for 1 min and resuspended in 400 μl PBS and PE- labeled antibodies against IgD. Flow cytometry was used to assess the subsets of B cells.

### Hematoxylin and eosin (HE) staining

Paraffin-embedded tissue sections (4 μm) from 3 randomly selected mice at each timepoint were dewaxed, hydrated and stained in hematoxylin solution for 3 min. The slices were then decolorized in acid alcohol (0.4% HCL in EtOH) for 15 s in hydrochloric acid ethanol differentiation liquid, washed in running tap water, and stained with eosin for 3 min. After sealed with neutral resin seal, they were examined under microscope.

### Luxol fast blue (LFB) staining

Paraffin-embedded tissue sections were soaked in ethanol: chloroform solution (1:1) for 5 min and 95% ethanol for 10 min, followed by overnight staining with 0.1% LFB solution at 56 °C. After staining, the slices were rehydrated through an ethanol serial and differentiated with 0.05% lithium carbonate solution for 5 min and 70% ethanol solution for 30 s. Well differentiated slices were stained with eosin for 1 min, washed with distilled water, and counterstained with 0.11% cresyl violet solution for 30 s. The slices were then cleared with xylene, sealed with neutral resin seal, and examined under microscope for changes in the myelin.

### Immunohistochemistry

Paraffin-embedded transverse tissue sections (4 μm) from 3 randomly selected mice at each timepoint were dewaxed, rehydrated. After washing in water, the slides were autoclaved for 3 min at 1.5 atm in sodium citrate buffer for antigen retrieval. Endogenous peroxidase activity was blocked with hydrogen peroxidase for 5 min at room temperature. After rinsing with tris buffered saline 1X (TBS), the tissue sections were incubated with primary antibodies at 4 °C overnight. The primary antibodies were antibodies against rabbit IL-4 (bs-0581R, Bioss), IL-17 (bs -1183R), IL2RA/CD25 (bs-0577R), IFN-gamma (bs-0480R), syndecan-1 (bs-1309R), CD27 (bs-2491R), CD19 (bs-4755R), CD4 (bs-0647R) and CD45 (bs-10599R), polyclonal antibody against CD3E (A1753, ABdonal) and plasma cell antibody[LIV3G11] (ab44876, abcam). The sections were subsequently washed with TBS 1X and incubated with secondary antibodies at room temperature for 30 min. Diaminobenzidine (DAB) and haematoxylin chromogen (Dako, Glostrup, Denmark) method was used to visualize the immunoreactions. For each treatment, images and mean optical density of positive cells (MOD) in 5 fields at high magnification were analyzed using ImagePro plus 6.0 image analysis software.

### Elisa

Tissues were homogenized and centrifuged to obtain supernatants. ELISA was performed using commercial kits according to the supplier’s instructions. All assayed were repeated at least three times. .

### Statistical analysis

All data were expressed as means ± standard error of the mean (SEM) obtained from at least three independent experiments. Statistical comparisons between experimental and control groups were assessed by using the Student’s *t*-test. *P* < 0.05 was considered statistically significant.

## Data Availability

The datasets used and/or analysed during the current study are available from the corresponding author on reasonable request.

## Abbreviations

CFA: Complete Freund adjuvant
DAB: Diaminobenzidine
EAE: Experimental autoimmune encephalomyelitis
ELISA: Enzyme linked immunosorbent assay
GADPH: Glyceraldehyde-3-phosphate dehydrogenase
HE staining: *He*matoxylin-eosin *staining*
MOG: Myelin oligodendrocyte glycoprotein
MS: Multiple sclerosis
PBS: Phosphate buffer saline
SEM: Standard error of the mean
TBS: Tris buffered saline
TPI: Triosephosphate isomerase
